# Recent advances in molecular targeted therapy of lung cancer: Possible application in translation medicine

**DOI:** 10.22038/IJBMS.2023.72407.15749

**Published:** 2024

**Authors:** Elnaz Salmani-Javan, Mahdi Farhoudi Sefidan Jadid, Nosratollah Zarghami

**Affiliations:** 1 Department of Clinical Biochemistry and Laboratory Medicine, Faculty of Medicine, Tabriz University of Medical Sciences, Tabriz, Iran; 2 Department of Medical Biochemistry, Faculty of Medicine, Istanbul Aydin University, Istanbul, Turkey

**Keywords:** Combination therapy, Immunotherapy, Lung cancer, Molecular targeted therapy, Translational medicine

## Abstract

Lung cancer is one of the leading causes of death among all cancer deaths. This cancer is classified into two different histological subtypes: non-small cell lung cancer (NSCLC), which is the most common subtype, and small cell lung cancer (SCLC), which is the most aggressive subtype. Understanding the molecular characteristics of lung cancer has expanded our knowledge of the cellular origins and molecular pathways affected by each of these subtypes and has contributed to the development of new therapies. Traditional treatments for lung cancer include surgery, chemotherapy, and radiotherapy. Advances in understanding the nature and specificity of lung cancer have led to the development of immunotherapy, which is the newest and most specialized treatment in the treatment of lung cancer. Each of these treatments has advantages and disadvantages and causes side effects. Today, combination therapy for lung cancer reduces side effects and increases the speed of recovery. Despite the significant progress that has been made in the treatment of lung cancer in the last decade, further research into new drugs and combination therapies is needed to extend the clinical benefits and improve outcomes in lung cancer. In this review article, we discussed common lung cancer treatments and their combinations from the most advanced to the newest.

## Introduction

A quarter of all cancer deaths are due to lung cancer and 82% of them are due to smoking. Almost 108,770 deaths from smoking due to lung cancer were reported in 2021, of which 3,590 were related to contact with secondhand smoke and causing 20,420 deaths from lung cancer. Therefore, smoking-induced lung cancer is one of the top ten causes of cancer death among men and women. But women in comparison with men have a higher portion of non-smoking lung cancer due to that they aren’t smokers like men. Hence the ratio of non-smoking lung cancer because of declines in smoking prevalence is increasing in both men and women (1). Lung cancer is classified into two major subtypes: non-small cell lung cancer (NSCLC) and small cell lung cancer (SCLC). NSCLC is the most general type of lung cancer compared with SCLC and represents almost 80–85% of lung cancer but SCLC is an invasive subtype and represents 5–10% of lung cancer (2). NSCLC and SCLC are different in genetic alteration and prognosis and require different treatment ways, thus this is important to consider the differences between these two subtypes during treatment (3). Based on the world health organization (WHO) NSCLC is classified into three main types: adenocarcinoma, squamous cell carcinoma, and large cell carcinoma, among which adenocarcinoma is the most usual type of NSCLC and responsible for around 40% of lung cancers. Also, squamous cell carcinoma is responsible for 25% to 30%, and large cell cancers account for 5% to 10% of lung cancers (4). According to recent studies, treatment and survival of NSCLC depends on its stage, for instance, surgical resection is recommended for stages I to II (5), but there is evidence about recurrent NSCLC and death in patients with resected NSCLC, and this event suggests that patients during surgical resection have micrometastasis (6).

**Figure 1:**
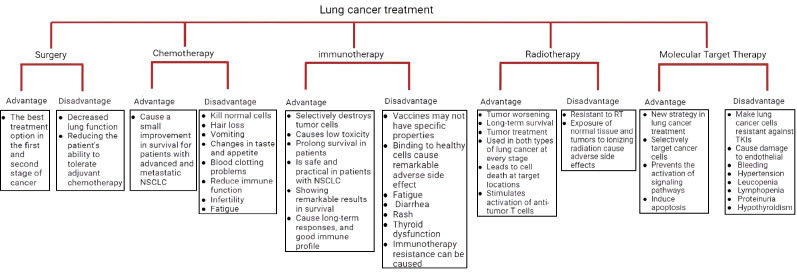
Advantages and disadvantages of current treatments in lung cancer

The main goal of the treatment of any cancer is to remove cancerous cells without destroying intact cells (7), and for this purpose various techniques such as the use of nanobubbles have been recommended (8, 9). According to recent research, modified drug-loaded nanobubbles improved drug targeting and accumulation within cancer cells, while decreasing drug accumulation within intact cells (10, 11). Treatment options for lung cancer are considered according to the stage of lung cancer, lung function, health status, and cancer features (12). According on recent studies much progress has been made in molecular target therapy and personalized therapy in the field of genomic alterations that prevent aberrant activation of signaling pathways, cellular growth, differentiation, and uncontrolled cell proliferation. In this review article, we have investigated various treatments and their application and advantages and disadvantages of current treatments in lung cancer which is shown in Figure 1.

**Surgery**

Surgery was once a common treatment for all types and stages of lung cancer (13) but nowadays surgery is recommended as the best therapy option in patients who are at the first and second stages of lung cancer. Also, surgery is essential in the treatment of multiple conditions in patients who are selected with stages III and IV (14). The best treatment option in patients with localized, mobile NSCLC and limited pulmonary metastases is surgery (15). The purpose of the surgery is to completely remove the primary tumor without any macroscopic tumor remaining. For this reason, patients with complete resection and without lymph node involvement are candidates for surgery (16). Patients with removable NSCLC that have simultaneous brain metastasis are treated with resection of brain metastasis after removal of the primary tumor of the lung. Research shows that the survival rate of lung cancer is 20.5% at 5 years (17). When the location of the primary tumor is controlled, removal of the metastatic lesion is recommended (14). Although surgery is a vital aspect of treating lung cancer, especially in the early stages of lung cancer, surgery alone has few results. About 30%–70% of patients who have lung resection experience recurrence or death (18). It is suggested that, at the time of surgery, a considerable ratio of patients have micro-metastatic disease (6).

Also after surgery, pulmonary function regardless of the surgical procedure declines (19) and it might delay patients’ ability to tolerate adjuvant chemotherapy (20). Hence many types of research have been done to find replacement treatments.

**Chemotherapy**

Although surgery is the recommended treatment in the early stage of NSCLC because a large number of patients with NSCLC are recognized in advanced stages and are not suitable for resection (21) chemotherapy can be helpful and causes small development in survival in patients with advanced stages and metastatic NSCLC (22, 23). Chemotherapy from the past till now is the main treatment that is used in the medical centers (24). In brief, chemotherapy is the usage of chemicals or drugs to destroy cancer cells (25), but chemotherapeutic drugs also kill normal cells and cause many side effects (21). These side effects include hair loss, vomiting, changes in taste and appetite, blood clotting problems, reduce immune function, and infertility (26) and among them, fatigue is the most common side effect (12). Even though most of the side effects after the chemotherapy are subsided but harm to the kidneys, heart, lungs, or the genital system sometimes is perennial (27). A significant problem in lung cancer therapy is resistance to chemotherapeutic agents which leads to disease progression and tumor recurrence (28, 29). A recent study has shown that a combination of chemotherapeutic agents could prevent or delay resistance to drugs. Also, the combination of more than two therapeutic agents has stronger antitumor effects in comparison with combinations of two agents (30). Besides, a recent study has shown that a drug combination treatment compared with a single drug treatment improves tumor markers such as CEA (21). However, these approaches are temporary, and using of multidrug agents eventually results in multi-drug resistance and this is the most important reason for defeat in lung cancer chemotherapy (31). Identifying the molecular mechanisms that cause resistance and developing efficient therapeutic strategies is important to overcome drug resistance (32, 33).

**Radiotherapy**

In cancer treatment, radiation therapy (RT) is an essential and effective part of the treatment and takes part in almost nearly all types of cancer and it can alleviate symptoms in patients with untreatable cancer (34). Ionizing radiation is used in RT because it can cause DNA damage that leads to cell death at target locations. Cancer cells divide in an abnormal manner and for this reason, they are more sensitive to radiation that induces DNA damage (35). Among tumor cells, undifferentiated cells have less ability to repair lethal DNA damage and are considered more susceptible to RT (36). Moreover, RT stimulates the activation of anti-tumor T cells which is dependent on induction type I interferon in the irradiated tumor (37). Exposure to RT was demonstrated to up-regulate and induce expression of MHCI in tumor cells and subsequently cause increase in intracellular peptide pool, antigen presentation, and identifying cytotoxic T lymphocyte of the irradiated cell. Immunotoxins such as perforin, granzymes, and granulin are produced by stimulated cytotoxic T cells, can penetrate the cytoplasm of the target cells, and lead to cell death. Ineffective T-cell migration is the major issue of immunotherapy that RT has been shown to overcome (38). 

As mentioned above RT is applicable in all stages of lung cancer. For patients who are in the early stages of NSCLC and are not suitable for surgery or are at high risk for operation stereotactic ablative radiotherapy (SABR) due to its limited toxicity, specially to old patients with poor lung function or other severe comorbidities it is recommended (39). SABR is the transference of large ablative doses of RT in a precision and accurate manner (40). SABR has mild toxicity and in most patients creates late radiological toxicity without severely impaired lung function (41). SABR creates a low risk of adverse side effects such as pneumonitis, chest pain syndrome, rib fractures for peripheral lesions, and damage to large arteries or airways (42). Like other treatments, several mechanisms make tumor cells resistant to RT. Recent studies have shown cancer stem cells and mutations in DNA damage-response pathways cause tumor cells to have radioresistance, genomic instability, and increase tumor heterogeneity, and among them, tumor heterogeneity plays a major role in radiation resistance (38, 43). As mentioned in the above section, the anti–PD-1 and anti–CTLA-4 combination with radiation can increase tumor-specific T cells in the draining lymph nodes. Also, it is represented that RT can have antitumor effects outside the radiation field (44). Recent studies have shown that the combination of RT and immunotherapy in NSCLC patients offers significant promise in expanding treatment options for patients (45). In addition, RT can be used alone or in combination with surgery or chemotherapy (34) and can cause tumor worsening, long-term survival, and even tumor treatment (46).

**Immunotherapy**

In the last few years, communication between the human immune system and cancer as cancer immunotherapy has developed significantly (47). Active immunotherapy is an evolving field in which immune modification is used to remedy cancerous malignancies (48). Immunotherapy has changed the treatment of many cancers, including lung cancer (49). There is evidence that if the immune system is stimulated properly, it can destroy cancerous cells (50). In contrast to chemotherapy which can swiftly reduce tumors, responses to immunotherapy are slow but lasting (51). In addition unlike other treatments, the main goal of immunotherapy is to prevent metastatic expansion and to improve the patient’s quality of life (52). Immunotherapy is based on recognition and tumor antigen presentation, inducing an immune response or blockage of immune cells for a better antitumor response. Immune response commences with antigen-presenting cells (APC) like dendritic cells, which transfer tumor antigens through major histocompatibility complex (MHC) molecules to T cells (53). Innate and adaptive immune systems have a major role in immune responses to cancer cells and APCs perform as bridges among innate and adaptive immune systems (54). Also, in non-immunogenic tumors, immunotherapy has generated significant interest. Although the immune system can devastate cancer cells and suppress tumor growth, it can also have a part in the expansion of tumors with a selection of tumor cells that can dodge supervision (55). The human body has an immune monitoring system that prevents the spread of tumor cells and destroys abnormal cells by the host immune system. Therefore escape from the immune monitoring system has a vital role in cancer expansion (52). Tumors utilize multiple mechanisms to evade the immune system. They do this by up-regulating the expression of molecules such as programmed death-ligand 1 (PD-L1), indoleamine-2,3-dioxygenase (IDO), siglec-9, down-regulating molecules like MHC class I, also through the expression of immune inhibitory cytokines including IL-10, tumor growth factor-beta (TGF-β), and prostaglandin E2 (PGE2), they begin to suppress the function of immune system cells, especially T cells, which are responsible for immune surveillance (56). Lung cancer cells have several immune suppression mechanisms which are essential for escaping the immune system. Lung cancer secret proteins such as STAT-3, indoleamine 2,3-dioxygenase (IDO), TGF-β, and IL-10 can prevent the routine process by APCs. Moreover, lung cancer cells block penetrating killer T cells by creating dense fibrotic stroma. A significant proportion of NSCLC has down-regulated MHC class I expression and has up-regulated Myeloid-derived suppressor cells (MDSCs). Up-regulation of MDSCs may be mediated by pro-inflammatory factors like PGE2, which inhibit T-cell function. Besides lung tumors inhibit activation of cytotoxic T-cell and natural kill (NK) cells by inducing abnormal proliferation of CD4+ FoxP3+ regulatory T cells (57). Immunotherapy is classified as passive or active.

***Active immunotherapy***

The main goal of active immunotherapy is to stimulate the host immune system, provide immune protection against tumor progression and development (58), and fortify the immune system of the host with specific tumor-associated antigens (TTA) or help cancer patients to develop an immune response against tumor cell devastation. The active immune system includes cancer vaccines, immune checkpoint inhibitors, and oncolytic viruses (59).

A tumor vaccine stimulates the immune system, and inducing immune responses against a defined tumor antigen is defined as an ideal candidate for therapy which can induce a preventive immune response. In addition, the induced immune response must be prolonged (52). One of the most important and basic features of tumor vaccines is that they must be detectable through the body’s antigens and recognized as alien by the host immune system. Viral vectors, proteins, naked DNA, peptides, whole cell-based vaccines such as autologous and heterologous, and dendritic cell (DC)-based vaccines are possible platforms where cancer vaccines can be prescribed (60). Autologous-dendritic cell vaccines are made from the host cancer cells used in the patients (61). DNA vaccines are used in conjunction with plasmid expression to create a target antigen (62). Vaccines based on vectors use viruses, bacteria, yeast cells, or other unique structures as carriers to transfer certain antigens to host cells (63). Allogeneic vaccinations contain antigens derived from non-cancerous cells. The tumor cells of other patients with the same tumor type are used to create antigens (64). The classification of some vaccines is mentioned in Table 1. 

Tumor vaccines are most efficient in patients with minimal disease such as after resection, definitive chemotherapy, or first-line combination chemotherapy (69). In lung cancer, autologous and allogeneic tumor cell vaccines have been studied. Autologous vaccines are specific for the patient: it needs the patient’s individual tissue to develop, and it might take weeks to months for the vaccine to be injected. Allogeneic vaccines require lung cancer cell lines but these tumor antigens may not have specific properties compared to the host tumor (65). Early reports on clinical examination using remedial vaccines in NSCLC have been disappointing, but right now vaccines are being identified with new targeted antigens and adjuvant therapies, and combining the vaccines with anti-PD-1/PD-L1 and anti-CTLA-4 drugs has given vaccines a new chance in cancer treatment (70). The Cytotoxic T-Lymphocyte Associated Protein-4 (CTLA4) and programmed cell death-1 (PD-1) checkpoint pathways are inhibitory pathways, and blocking these inhibitory pathways with mAbs has triggered antitumor immune responses that altered the cancer therapy field. Recent studies have shown dual blockade of PD-1 and CTLA-4 inhibitory pathways, remarkably enhances anti-tumor immune responses (55). Also targeting the PD-1, programmed cell death ligand 1 (PD-L1), and CTLA4 pathways, demonstrate significant advances in lung cancer treatment (53). Immune cells like T cells, NK, and MDSCs, after their activation, express the PD-1 receptor, with major role in the limitation of T cells activity in peripheral tissues. Different types of tumors, including NSCLC, highly express PD-L1 on their surface suggesting that activation of the PD-1 / PD-L1 pathways is a usual mechanism that tumor cells use to evade immune monitoring and growth. Recent preclinical studies have shown that PD-1 signal blocking can restore the cytotoxic functions and capabilities of T + CD8 cells from exhausted phenotype and increase anti-tumor immunity. Moreover, monoclonal antibodies can block PD-L1 and inhibit PD-1 from reacting through PD-L2 and CD80 which appears to control inflammation and protect intact lung tissue from further injury while the immune system is activated. In lung cancer, anti-PD-1 and anti-PD-L1 monoclonal antibodies have demonstrated considerable activity, showing remarkable results in survival, long-term responses, and good immune profile compared to cytotoxic chemotherapy (71). In advanced NSCLC several examinations have been reported with treatment with anti-PD-1 / PD-L1 antibodies, alone or in combination with chemotherapy (72). 

Like PD_1, CTLA-4 is a surface receptor (73) that is expressed on the surface of T cells and regulates the activation of T cells, moderates T helper cells activity, and increases the immunosuppressive activity of regulatory T (Treg) cells (74). Based on studies lung cancer cells can stimulate abnormal CTLA-4 expression in T cells, so lung cancer cells might cooperate with the CTLA-4 pathway to escape from T cells. In several cancers inhibition of CTLA-4 with monoclonal antibody get consistent and lasting antitumor responses (73). Data from recent studies suggest that targeting CTLA-4 and Treg cells with immunotherapy may have an advantage in lung cancer patients (75). However, blocking the PD-1 pathway, compared with blocking CTLA-4, has more therapeutic activity and restricted toxicity, maybe because of the chronically stimulated state. Since the advantage of monotherapy is limited due to low rate responses and just a subset of patients responds to treatment (76). dual anti-CTLA-4 and anti-PD-L1 Anti-CTLA-4 and anti-PD- (L) 1 are much investigated in NSCLC (77). According to a recent study, CTLA-4 and PD-1 blockers combination is effective and increases response and survival in several types of cancer including lung cancer (76).

Furthermore, the combination of immunosuppressive inhibitors and chemotherapy has recently been revealed to have improved the survival rates in patients with lung cancer through several mechanisms such as improvement of antigen delivery to T cells and Removal of immunosuppressive components of the tumor immune microenvironment (78).

Oncolytic viruses (OVs) are new therapeutic agents in the field of cancer treatment (79) due to their capability to keep normal cells healthy while selectively infecting and destroying cancer cells (80). The antitumor activity of OVs is mediated through two different mechanisms of action: selective proliferation in neoplastic cells, which results in a direct lytic effect on the tumor cell and induction of systemic antitumor immunity. Based on the nature and type of tumor cells and OVs, the interaction between the virus, tumor environment, and host immune system, OVs mechanism of action may be different (81). Nowadays, adenoviruses, herpes viruses, measles viruses, coxsackie viruses, polioviruses, reoviruses, poxviruses, and Newcastle disease viruses are OVs that have been developed for the treatment of cancer (82). Recent studies have demonstrated that a combination of OVs with anti-PD1 therapy elicits a high response rate in patients with advanced melanoma (83).

OVs are an attractive treatment for lung cancer treatment due to the transmission of therapeutic viral particles through the intranasal pathway. Genetically altered oncolytic viruses (OVs) can destroy tumor cells through unique mechanisms and can be used to transport toxic, therapeutic, or immune-regulating genes to tumor cells. In addition, OVs are a promising approach to overcoming drug resistance in lung cancer, such that the use of highly immunogenic OVs, to establish consistent immunity during viral delivery of drug-resistant antigens, leads to increased antitumor immunity (84). 

***Passive immune therapy***

Unlike active immune therapy which depends on the host immune system’s ability to produce a specific immune response to tumor antigen, passive immune therapy administers exogenous lymphocytes or antibodies to interfere with the immune responses and target specific tumor antigens (59, 85). On the other hand, passive immune therapy uses elements of the immune system, which are produced outside the body, to boost anticancer responses. While the patient’s immune response is fragile and unable to respond, the use of passive immunotherapy becomes essential to compensate for the patient’s immunological deficiency (86). Passive immune therapy includes monoclonal antibody (mAb) and adoptive T cell transfer (59). MAbs are classified in passive-specific immune therapy and adoptive T cell transfer in passive non-specific immunotherapy (87). 

MAbs belong to single-cell clones which are produced by cells of the immune system against a specific antigen and specifically bind to it. Receptors such as epidermal growth factor receptor (EGFR) and receptor for a vascular endothelium growth factor (VEGF) have a role in tumor progression, and when mAbs are injected inhibit their effect (87). MAbs therapy is not as toxic as chemotherapy, however, in some cases binding to healthy cells causes remarkable adverse side effect (88). Some mAbs are approved to be used against these receptors in NSCLC (89).

As mentioned above, adoptive T cell immune therapy is another type of passive immune therapy, in which immune T cells are extracted from the patient’s body and after activation, modification, and proliferation *in vitro*, are transferred back to the patient’s body as treatment (91). Recently adaptive T-cell therapy results were encouraging in cancer treatment. In addition, a recent study has shown adaptive T-cell therapy is safe and practical in patients with NSCLC (91). Unfortunately, while the expression of tumor antigens decreases or is lost, cancer cells become resistant to passive immune therapy (92).

By contrast, specific passive immunotherapy triggers certain immune responses to specific tumor antigens, and nonspecific passive immunotherapy creates general responses. In general in passive immune therapy, the anti-tumor effect is temporary and immunological memory is not generated (93). Responses from immune therapy are not stable and only a minority group of patients have extended benefits. Immunotherapy resistance can be caused by various mechanisms including change in the tumor microenvironment (TME) or the presence and persistence of a resistant clone and it may not be resolved simply by the ligand or receptor inhibition (49). The most common adverse side effects of immune therapy include fatigue, diarrhea, rash, and thyroid dysfunction (94). Also when two immunotherapeutic agents are combined, the side effects of immunity worsen (95). When it comes to comparing immune therapy with chemotherapy, immune therapy, unlike chemotherapy, selectively destroys tumor cells and causes low toxicity. induction of immunological memory can overcome resistance to chemotherapy and thus may delay relapse and prolong survival in patients (96). In general in lung cancer, immunotherapy has promising effects (95).

**Molecular target therapy**

**Figure 2:**
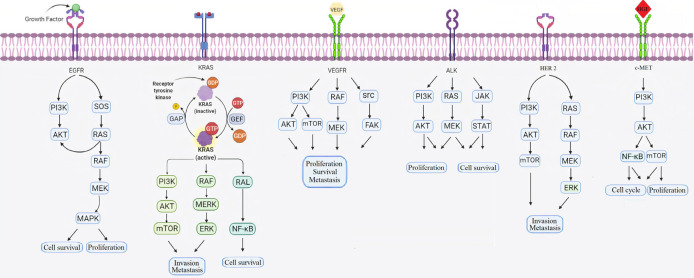
Signaling pathways are involved in the proliferation and survival of lung cancer cells

Molecular target therapy is evolving therapy that interacts with particular molecules to stop the development, progression, and tumor metastasis, (97, 98) and causes high effectiveness and less toxicity than traditional treatment. Recent studies have revealed that molecular target therapy has led to significant clinical success in the treatment of numerous cancers like chronic myelogenous leukemia, colon cancer, breast cancer, and lung cancer (99). The use of molecular target therapy has improved the survival rates of lung cancer patients (97). Drugs that are used in molecular target therapy, block important signaling pathways that lead to cancer progression (100). EGFR, ALK, VEGF, and KRAS are the most important signaling pathways which have been identified and are involved in the progression of lung cancer (Figure 2).

***Epidermal growth factor receptor tyrosine kinase***

*Function and dysfunction*

 EGFR is encoded by the EGFR gene (101) and is widely expressed in normal tissues originating from epithelial, mesenchymal, and neural cells (102). EGFR is a kind of transmembrane protein that has cytoplasmic kinase activity. EGFR activates downstream signaling pathways such as RAS-MAPK, PI3K-AKT, STAT, and binding of growth factors (epidermal growth factor) stimulates these signaling pathways, whose most important obligation is transferring important growth factor signaling from the extracellular environment to the cell (103, 104). Activation and regulation of EGFR and downstream genes lead to apoptosis, proliferation, and angiogenesis (105). Mutations in EGFR activate the abnormal signaling pathway and cause an increase in cell survival, proliferation, angiogenesis, and a tendency to metastasize (106). It is observed that EGFR is expressed almost in 93% of NSCLC patients, of which about 45% are overexpressed (107) and mostly found in patients who are not smokers (108). For this reason, EGFR acts as a tumor marker and becomes an attractive tool in molecular lung cancer target therapy (109). High expression of this gene has been attributed to treatment resistance and poor diagnosis (106). However, lung cancer patients, who have mutations in the EGFR gene, are sensitive to tyrosine kinase inhibitors (TKIs) drugs (110). 

*Therapeutic application*

TKIs against EGFR mutations are classified into first, second, and third generations (111). EGFR-TKIs have achieved significant clinical success in recent years, but despite these successes, resistance to treatment has been observed in special EGFR mutations like C797S and T790M (112). T790M increases the affinity of ATP and ATP binding site and prevents drug binding (113). Drug resistance with the EGFR T790M mutation was observed in 50% of patients treated with first- and second-generation EGFR-TKIs (114). Osimertinib is a third-generation EGFR-TKL that selectively and significantly suppresses T790M-resistance mutations in lung cancer patients and is approved in the first-line treatment of EGFR-mutant lung cancer (113, 114). Latest studies have demonstrated that targeting the ATP binding pocket of EGFR with the small molecule can be a hopeful new strategy in lung cancer treatment. Monoclonal antibodies and TKIs are two classes of EGFR inhibitors that are used in clinical treatment (7). There are monoclonal antibodies that are against the extracellular domain of EGFR, for example, one is stoxymab, which is made to treat patients with lung cancer (115). There are three generations of TKIs that are used in lung cancer treatment. First-generation agents including gefitinib and erlotinib, bind to EGFR in a competitive and reversible way. Second-generation agents such as Afatinib, covalently bind to the kinase domain of EGFR (116). Third-generation agents include osimertinib, which selectively and irreversibly inhibits the T790M EGFR mutant. But unlike the previous two generations, the third-generation TKIs, have little effect on wild-type EGFR and selectively target EGFR harboring T790M. Thus, third-generation TKIs overcome the toxicity limitations seen in the first and second generations (117, 118). Manifestation of secondary mutations such as T790M and C797S, stimulation of another signaling pathway like Met, downstream pathway deviation like AKT mutations, and EGFR-TKIs-mediated apoptotic pathway disruption are the mechanisms that make lung cancer cells resistant to TKIs (109). Therefore, more detailed information on the signaling mechanism and pathway is needed to understand and overcome EGFR-TKI resistance.

***Vascular endothelial growth factor***

*Function and dysfunction*

VEGF binds to VEGF receptors on vascular endothelial cells which regulate vascular endothelial cell proliferation and migration, stimulate angiogenesis in embryonic growth, and heal adult wounds (119, 120). The effects of VEGF on cells through several signaling pathways including the PI3K pathway, MAPK / ERK pathway, and others (120). Tumor cells in order to keep growing and survive, need the formation of new blood vessels. This process is provided through the angiogenesis process, and for this reason, angiogenesis becomes a hallmark of cancers (121). Tumor tissue vessels are aberrant, twisting, fragile, and leaky. Due to the detachment of perivascular cells, tumor vessels are incoherent, immature, dysfunctional, and less integrated, and this feature increases tumor spread and metastasis (122). VEGF is a major mediator of tumor angiogenesis that causes stimulation of the growth of new blood vessels and provides tumor cells oxygen and nutrients (119). Moreover, recent data have shown that VEGF is involved in tumor metastasis. This suggests that VEGF and its signaling pathways appear as attractive targets for the treatment of various types of cancer, including lung cancer. There are reports that revealed serum levels of VEGF are high in both types of lung cancer. VEGF through activation of MEK/ERK and PI3K/AKT signaling pathways induces lung cancer cell proliferation (123, 124). In lung cancer treatment, targeting VEGF with antibodies and VEGF receptors with small molecules has been studied (106).

*Therapeutic application*

Anti-VEGF monoclonal antibodies, such as bevacizumab, inhibit the VEGF signaling pathway by binding to and neutralizing all human VEGF isoforms and proteolytic components in lung cancer. But since the inhibition of VEGF with bevacizumab did not inhibit lung cancer cell growth, it was found that VEGF alone could not maintain the proliferation of lung cancer cells (123). VEGFR-TKIs are another type of anti-VEGF that prevents the activation of signaling pathways, thereby preventing the growth of blood vessels. The combination of VEGF-TKI with chemotherapy causes the destruction of tumor cells and the rapid shrinkage of the tumor. The latest studies, however, have shown that combining VEGF-TKI with chemotherapy in lung cancer may not provide overall survival, although it has shown a higher degree of toxicity (125). As discussed above, the EGFR signaling pathway is deregulated in cancers, and EGFR and VEGF signaling has a major role in angiogenesis, tumor development, and metastasis, and dual inhibition of these signaling pathways is considered a potential strategy of lung cancer treatment (126). Anti-VEGF drugs reduce the oxygen supply to tumor cells and trigger hypoxia but these effects stimulate cancer cells to express alternative angiogenic proteins and create resistance. Drugs, which dually inhibit EGFR and VEGF pathways, postponed treatment resistance (127). Due to VEGF triggering angiogenesis in healthy tissues, using anti-VEGF drugs causes side effects, toxicities, damage to endothelial cells, bleeding, hypertension, leucopenia, lymphopenia, proteinuria, and hypothyroidism (121). Therefore, it should be considered that drugs should be designed that have the least side effects. 

***Anaplastic lymphoma kinase***

*Function and dysfunction*

ALK gene encodes ALK, which is a receptor tyrosine kinase, involved in the development of the nervous system during embryogenesis. In adult persons, only specific neurons express ALK. RAS–MAPK, PI3K –AKT, and JAK-STAT pathways are regulated by ALK (128). Mutation, gene amplification, and chromosomal rearrangement are the reasons that abnormally activate ALK (129). The most usual abnormality of ALK that occurs in lung cancer is ALK rearrangement so 3% to 5% of lung cancer patients have ALK rearrangement (130). Translocation between the N-terminal of echinoderm microtubule-associated protein-like 4 (EML4) and ALK gene leads to EML4-ALK formation (131) which has carcinogenic and malignant features (132) and occurs in 80% of ALK positives in lung cancer. ALK translocation increases tyrosine kinase activity, which increases cell proliferation, survival, and tumorigenesis (133). Also, ALK fusion is used as a therapeutic target that responds well to ALK TKI (134) which leads to inhibiting the ALK downstream signaling pathway and induces apoptosis (135, 136). 

*Therapeutic application*

There are three generations of ALK inhibitors: the first generation of ALK inhibitor include crizotinib, the second generation include ceritinib, and the third generation include lorlatinib (105). One of the second-generation ALK inhibitors is alectinib which is efficient for ALK mutations and rearrangement (137). ALK autophosphorylation and phosphorylation of STAT3 are inhibited by aletcinib, also aletcinib could suppress the development of EML4-ALK-positive tumor cells (138). Like other therapies, there are mechanisms that make tumor cells resistant to ALK inhibitors. Different types of integration of EML4 with ALK or other genes (133), the presence of a secondary mutation in ALK, and activation of alternative signaling pathways such as EGFR, are the mechanisms by which tumor cells become resistant (139). For a better and more targeted treatment, combination therapies with other targeted agents and the combination of ALK inhibitors with immunotherapy require more clinical and clinical research (105). 

***KRAS (Kirsten rat sarcoma virus)***

*Function and dysfunction*

 KRAS proto-oncogene gene encodes the KRAS protein, a guanine triphosphatase (GTPase)*,* which has a major role in the regulation of several cell functions and acts as signal transduction for EGFR, MET, and ALK (140). KRAS activity is controlled by the GTP/GDP ratio (141). The most common mutation in KRAS is point mutation (108) which inhibits KRAS ability in the hydrolysis of GTP and activates KRAS downstream signaling cascades, leading to uncontrolled cell proliferation and survival (142). Deregulation of the KRAS pathway is found in 25% of NSCLC cases (143). The G12C mutation, which is present in 16% of all lung adenocarcinomas, is the most frequent change in KRAS in lung cancer (144). The KRAS G12C mutation has been identified as a possible target for new therapeutics (145). 

*Therapeutic application*

Two powerful, specific, and irreversible small-molecule KRASG12C inhibitors, adagrasib and sotorasib, have shown encouraging outcomes in NSCLC treatment (146). In resistance to EGFR TKI and monoclonal antibodies, KRAS activation plays a major role because, despite the inhibition of EGFR with TKIs, KRAS activation permits EGF-mediated downstream signaling (147). Also, The KRAS mutation is associated with cellular and clinical radiographic resistance (148). Three strategies were considered to directly inhibit KRAS activity. First, creating a competitive repressor to stop the formation of GTP-KRAS, second, improving the GTPase activity of mutant cells with KRAS, and third, inhibiting the activation of KRAS by targeting its membrane binding via phosphodiesterase (143). Most patients are usually treated with a combination of chemotherapy with immunotherapy or immunotherapy alone (141). Recent study has shown that cells with KRAS mutation, are sensitive to inhibition of MEK, IGF-and mTOR signaling pathways (144). Also, previous studies showed that direct inhibition of RAS activation has no clinical effect, but inhibition of targets downstream of the mitogen-activated protein kinase (MEK) pathway may be an encouraging strategy (143).

***Human epidermal growth factor 2***

*Function and dysfunction*

 HER2 gene encodes the tyrosine kinase receptor of the ERBB family which directly adjusts the EGFR signaling pathway that leads to activation of MAPK, JAK-STAT, and P3K/Akt signaling pathways (145). In several malignancies like bladder, breast, ovarian, stomach, pancreatic, and lung cancers, the HER2 signaling pathway is hyper-activated which results in uncontrolled cell development (149). Overexpression, amplification, and mutation are three types of HER2 gene aberration observed in NSCLC (150). Based on several recent studies, in NSCLC patients HER2 overexpression is linked to poor outcomes while the predictive importance of HER2 mutation and amplification is unknown (149). 

*Therapeutic application*

Chemotherapy is one of the most important therapeutic agents for patients with HER2-altered lung cancer, HER2-positive tumors are resistant to chemotherapy (151). This feature has led HER2-positive tumors to become candidates for molecular target therapy (152). Numerous Abs and HER2-targeted tyrosine kinase inhibitors have been studied for HER2-positive tumor treatment. However, responses to HER2-targeted tyrosine kinase inhibitors such as afatinib, lapatinib, and neratinib were not satisfactory (151). Although anti-HER2 drugs were effective in the treatment of gastric and breast cancer, they did not seem to work well in the treatment of lung cancer (153). As a result, more therapeutic options for NSCLC patients with HER2 mutations are required (151).

***Raf murine sarcoma viral oncogene homolog B***

*Function and dysfunction*

The BRAF gene encodes a serine/threonine-protein kinase and belongs to the Raf kinase family that regulates cell development, differentiation, and proliferation through the MAPK signaling pathway (154, 155). Mutations in the BRAF gene will result in the development and progression of cancer (156). BRAF mutations, mostly as a V600E mutation, have been discovered in 50% of all melanomas (155). BRAF is one of the most critical genes linked to the development of NSCLC and is found in 1.5-3.5% of NSCLC patients (157, 158).

*Therapeutic application*

 Based on conducted studies NSCLC patients with V600E mutations are resistant to chemotherapy (156). Immune checkpoint inhibitors have little efficacy in BARA-mutated NSCLC patients thus BRAF target therapy is the present choice of treatment in BARA-mutated NSCLC patients (156). BRAF inhibitors, either alone or in combination with MEK pathway inhibitors, are another efficient therapeutic option for BRAF-mutated NSCLC, with a better response rate (154). BRAF inhibitors have shown to have promising therapeutic benefits, however, they are only effective for a short time since approximately all patients develop resistance to the treatment within a few months. Furthermore, the causes of resistance to BRAF-targeted treatment in NSCLC patients remain unknown, which limits the development and deployment of similar targeted treatment methods (158).

***C-ros oncogene***

*Function and dysfunction*

ROS proto-oncogene encodes a membrane protein with tyrosine kinase activity. ROS1 has a critical role in the activation of JAK/STAT, RAS/RAF/MEK/ MAPK, and PI3K/AKT/mTOR pathways activation of which causes cell development, proliferation, and cell differentiation. Any alteration in the ROS1 gene contributes to tumor formation and progression (159). In numerous types of cancers such as ovarian and colorectal cancer ROS1 gene rearrangement has been found. ROS1 rearrangements are seen in about 1–2% of NSCLC patients who are young, female, and have never smoked (160). ROS1 is currently recognized as a unique molecular target in NSCLC (161). 

*Therapeutic application*

ROS1 can be treated by applying a combination of treatments like surgery, radiotherapy, immunotherapy, chemotherapy, and target therapy (162). As discussed above, ROS1 belongs to the tyrosine kinase family and for this reason, TKIs are the first-line treatment option in patients with ROS1 rearrangement (163). Recent clinical study data have shown that Crizotinib, which is TKI, approved by FDA and used as a target therapy drug, can inhibit ROS1. As the administration of ROS1 inhibitors becomes more common, precise and early identification of ROS1 gene rearrangements will be crucial for patients with NSCLC to get the best possible therapy (161, 162).

***C-mesenchymal-epithelial transition factor***

*Function and dysfunction*

c-MET gene produces a tyrosine receptor kinase that has an essential role in vital biological functions including cell development, cell cycle, cell differentiation, repair of injured tissues, liver regeneration, and embryogenesis (164, 165). c-MET and its ligand hepatocyte growth factor (HGF) activate MAPK and PI3K/AKT/mTOR signaling pathways. In numerous malignancies, including NSCLC, up-regulation, amplification, or mutation of the c-MET receptor causes carcinogenesis, poor prognosis, and metastasis. As a result, it has been considered an interesting therapeutic anti-cancer target(166, 167). According to several studies, c-MET overexpression was identified in 60% of NSCLC patients (168). 

*Therapeutic application*

Current treatments include receptor targeting and ligand binding, TKIs of c-MET, and antibodies against c-MET or HGF (1169). A recent study has shown that decreasing the expression of c-MET lowers cell growth and survival. Moreover, c-MET suppression prevents HGF-induced EMT(170). Applying immunotherapy for NSCLC patients with c-MET modifications could be prescribed after chemotherapy and target therapy but this should be explored more (165).

**Table 1:** Types of vaccines developed to treat a variety of cancers

Cancer vaccines	Source of cancer vaccine	Ref
Autologous-dendritic cell vaccines	Such vaccines are prepared from the same patients' cancer cells that are used.	(61)
DNA vaccines	These vaccines are combined with a plasmid expression that constitutes a target antigen.	(62)
Vector-based vaccines	These vaccines contain viruses, bacteria, yeast cells, or other special structures as carriers that can be used to deliver specific antigens to host cells.	(65)
Allogeneic vaccines	The sources of antigens in these vaccines are from non-cancerous cells. Antigens are constructed from the tumor cells of other patients with identical types of tumors.	(64)
Whole-cell vaccines	These vaccines use whole cancer cells (either live or dead) that have been modified in the laboratory to make them more visible to the immune system.	(66)
Peptide vaccines	These vaccines use small pieces of proteins from cancer cells to stimulate an immune response.	(67)
Dendritic cell vaccines	As previously mentioned, these are personalized vaccines that use the patient's own dendritic cells to activate the immune system against cancer cells.	(68)
mRNA vaccines	These vaccines use messenger RNA (mRNA) molecules that encode proteins found in cancer cells to stimulate an immune response.	(69)

Activation of each of these signaling pathways activates PIK3, RAF, and RAS, which are directly involved in the progression and development of lung cancer.

## Conclusion

As cancer is a heterogeneous disease involving genetic, architectural, metabolic, pathophysiological, and immunological complexities many attempts have been made to identify biomarkers associated with innate and acquired radioresistance. In both types of lung cancer, early diagnosis and treatment in the early stages of the sickness are basic and the most important condition for effective therapy, therefore it is important to focus on increasing early detection of lung cancer. Monotherapy is not particularly effective in treating cancers, and there have been significant efforts to develop optimal combination methods to improve the efficacy and therapeutic effects of anticancer therapy. 

In summary, we have reviewed an update for molecular target therapy of lung cancer and the advantages and disadvantages of new combination therapy for lung cancer which could pave the way for efficient lung cancer therapy.

## Authors’ Contributions

E S wrote the original draft; M FSJ edited the manuscript; N Z conceived and supervised the study. All named authors meet the International Committee of Medical Journal Editors (ICMJE) criteria for authorship of this article, take responsibility for the integrity of the work as a whole, and have given their approval for this version to be published. 

## Funding

The authors declare that no funds, grants, or other support were received during the preparation of this manuscript.

## Compliance with Ethics Guidelines

This article is based on previously conducted studies and does not contain any new studies with human participants or animals performed by any of the authors.

## Conflicts of Interest

No potential competing interests were reported by the authors.
